# Downregulation of TET1 Promotes Glioma Cell Proliferation and Invasion by Targeting Wnt/*β*-Catenin Pathway

**DOI:** 10.1155/2021/8980711

**Published:** 2021-12-09

**Authors:** Jianwen Ji, Qiuxiang You, Jidong Zhang, Yutao Wang, Jing Cheng, Xiangyun Huang, Yundong Zhang

**Affiliations:** Department of Neurological Center, The Third Affiliated Hospital of Chongqing Medical University (General Hospital), Chongqing 401120, China

## Abstract

Glioma is the most common malignant tumor in adult brain characteristic with poor prognosis and low survival rate. Despite the application of advanced surgery, chemotherapy, and radiotherapy, the patients with glioma suffer poor treatment effects due to the complex molecular mechanisms of pathological process. In this paper, we conducted the experiments to prove the critical roles TET1 played in glioma and explored the downstream targets of TET1 in order to provide a novel theoretical basis for clinical glioma therapy. RT-qPCR was adopted to detect the RNA level of TET1 and *β*-catenin; Western blot was taken to determine the expression of proteins. CCK8 assay was used to detect the proliferation of glioma cells. Flow cytometry was used to test cell apoptosis and distribution of cell cycle. To detect the migration and invasion of glioma cells, wound healing assay and Transwell were performed. It was found that downregulation of TET1 could promote the proliferation migration and invasion of glioma cells and the concomitant upregulation of *β*-catenin, and its downstream targets like cyclinD1 and c-myc were observed. The further rescue experiments were performed, wherein downregulation of *β*-catenin markedly decreases glioma cell proliferation in vitro and in vivo. This study confirmed the tumor suppressive function of TET1 and illustrated the underlying molecular mechanisms regulated by TET1 in glioma.

## 1. Introduction

As shown in previous studies, modification of DNA plays critical roles in cell proliferation, apoptosis, tumorigenesis, and metastasis [1, 2]. Methylation at 5-position of cytosine (5mC) is the most common DNA modification in CpG dinucleotide which has regulatory function on expression of genes and genomic stability [3, 4]. It is proved that demethylation of 5mC can be converted into 5-hydroxymethylcytosine (5hmC) mediated by ten-eleven translocation (TET) family, TET1~TET3 [5, 6].

5hmC is abundant in nervous system, and it has been found in a variety of nerve cells such as neural stem cells, neurons, astrocytes, and Purkinje cells [7, 8]. Expression level of 5hmC plays critical roles on neurogenesis, differentiation, and neurodevelopment in embryonic phase and adults. For example, Kim et al. determined that overexpression of TET1 promotes neurogenesis ahead of time during the development of fetal brain in mice [9]. Li et al. prove that overexpression of TET2 in the hippocampus of mature adults can enhance the expression of 5hmC and promote the neurogenesis in hippocampus which is declined by ageing and improve the ability of learning and memory [10].

Amount of studies suggest that 5hmC and TET1 play significant roles in cancer process. It is proved that TET1 can act as a tumor suppressor in colon cancer because downregulation of TET1 can promote the expression of oncogene axis inhibition protein 1 and cell proliferation in colon cells by targeting *β*-catenin pathway [11]. In osteosarcoma cells, overexpression of TET1 can inhibit cell proliferation and induce cell apoptosis by unregulated 5hmC levels [12]. In addition, TET1 is found downregulated in glioma specimens, and downregulation of TET1 facilitates proliferation and invasion in glioma cells by inhibiting the cellular autophagy [13].

In accordance with previous studies, our study demonstrates that TET1 is downregulated in malignant glioma (WHO grade IV glioma) specimens, and knock down of TET1 promotes proliferation, migration, and invasion of malignant glioma cells. Results of xenografting prove that downregulation of TET1 inhibits tumor growth in vivo. Furthermore, we also conduct experiments to explore molecular mechanisms mediated by TET1 in tumor growth. It is found that downregulation of TET1 inhibits the expression of *β*-catenin which is one of the key regulators of Wnt/*β*-catenin pathway, and the further rescue experiments confirm the regulatory function of TET1 on Wnt/*β*-catenin signaling pathway.

## 2. Material and Methods

### 2.1. Patients

Tumor tissues and the adjacent normal tissues were obtained from 24 patients with malignant glioma who received surgery in The Third Affiliated Hospital of Chongqing Medical University (General Hospital). All patients did not received any other therapy before surgery. Informed consent was provided by all patients or their family. This study is in line with the Declaration of Helsinki and approved by the Ethics Review Committee of The Second Affiliated Hospital of Army Medical University (Ethical Lot Number: CQ-011-201568652).

### 2.2. Cell Culture

Human glioma cell lines U251 and SW1783 cells were purchased from American Type Culture Collection (ATCC, VA, USA). U251 and SW1783 cells were cultured with Roswell Park Memorial Institute 1640 medium (RPMI-1640, Thermo, NYC, USA) with 10% fetal bovine serum (FBS, Gibco, CA, USA) in incubator at 37°C, 5% CO_2_.

### 2.3. Cell Transfection

Negative control shRNA, TET1 shRNA, and *β*-catenin shRNA were transfected into U251 and SW1783 cells with Lipofectine 2000 (Invitrogen, CA, USA) according to manufacturer's instruction.

### 2.4. Real-Time Quantification PCR

Total RNA of U251 and SW1783 cells was extracted with Trizol regent (Thermo, NYC, USA). RNA was reverse trancripted into cDNA with QuantiTect Reverse Transcription Kit (Qiagen, NY, USA). SuperRT One Step RT-PCR Kit (CWBIO, Beijing, China) was taken to detect the expression of TET1, *β*-catenin, cyclinD1, and c-myc. GAPDH was used as internal control. Primers used for PCR are shown as follows: TET1-forward: 5′-TCTGTCCTGGGGAGACACTG-3′, TET1-reverse: 5′-GAGTCGGTTCCCAAAGGTCC-3′; *β*-catenin-forward: 5′-GAAGGGGCTGACGCTATTGA-3′, *β*-catenin-reverse: 5′-GCTCCCAAGGAACGTCATCA-3′. cyclinD1-forward: 5′-ACATGGTGGATGAAGTGGACA-3′, cyclinD1-reverse: 5′-CAAGTGTCCAGAAGGTGTGAC-3′; c-myc-forward: 5′-TTTGCGTCGCCAGGTGAAGA-3′, c-myc-reverse: 5′-GTGTGACCTTGTTTCACTTCCG-3′. GAPDH-forward: 5′-TTTGCGTCGCCAGGTGAAGA-3′, GAPDH-reverse: 5′-AGTTAAAAGCAGCCCTGGTGA-3′.

### 2.5. Western Blot

U251 and SW1783 cells were lysed by lysis buffer with RIPA on ice for 30 min and centrifuge at 4°C for 20 min at 12000 g/min. The supernatant was transferred into another microphage tube and quantitated with BCA Protein Assay Kit (Bio-Rad Laboratories, CA, USA). Protein samples were separated with SDS-polyacrylamide gels and transferred to polyvinylidene difluoride (PDVF) membranes (Millipore, MA, USA). After being incubated with 5% BSA block buffer, the primary antibody was used as follows: rabbit anti-TET1 (ab220867, Abcam, MA, USA), rabbit anti-cyclinD1 (ab16663, Abcam, MA, USA), rabbit anti-c-myc (ab32072, Abcam, MA, USA), rabbit anti-*β*-catenin (ab32572, Abcam, MA, USA), mouse anti-GS3K*β* (ab93926, Abcam, MA, USA), mouse anti-p-GSK3*β* (ab68476, Abcam, MA, USA), GAPDH (Santa Cruz Biotechnology, TX, USA), and the membrane was detected by chemiluminescent ECL reagent (Bio-Rad Laboratories, CA, USA).

### 2.6. CCK8 Assay

Cells transfected with TET1 shRNA were seed on 96-well plates and cultured with 100 *μ*l cell culture medium. After culturing for 48 hours, 10 *μ*l CCK8 was added into cell culture medium and incubated at 37°C for 2 h. Calculate the absorbance at 450 nm with a plate reader (model 680; Bio-Rad, Hertfordshire, UK).

### 2.7. Wound Healing Assay

Cells transfected with TET1 shRNA and *β*-catenin shRNA were seeded into 6-well plates in density of 1 × 10^5^ cells/well and cultured for 48 h; the wound was scratch with 20 *μ*l pipette. Images were taken at 0 h and 24 h; the width was measured with ImageJ.

### 2.8. Transwell Experiments

The upper chambers were coated with Matrigel, and the cells transfected with NC shRNA, TET1 shRNA, and *β*-catenin shRNA were seeded in the density of 1 × 10^5^ cells/well, and the upper chamber was added with 200 *μ*l medium without serum. In the lower chamber, 600 *μ*l cell culture medium with 15%FBS was added. Culture the cells at 37°C for 48 h, and the invasive cells were fixed with 4% paraformaldehyde (PFA) staining with crystal violet.

### 2.9. Colony Formation Assay

Cells transfected with NC shRNA and TET1 shRNA were seeded into 6-well plates in a density of 100 cells/ml. After 14 d culturing, the cells were fixed with 4% PFA and stained with crystal violet.

### 2.10. Cell Apoptosis Assay

Cells transfected with NC shRNA and TET1 shRNA were collected with 0.25% trypsin and washed with phosphate buffer saline for three times. After that, cells were costained with Annexin V-FITC/PI and detected by flow cytometry.

### 2.11. Cell Cycle Distribution Assay

Cells transfected with NC shRNA and TET1 shRNA were collected with 0.25% trypsin and washed with phosphate buffer saline for three times. After that, fix the cells with 70% ethanol for 3 h. PI was used to stain the cells, and flow cytometry was performed to detected the distribution of cell cycle.

### 2.12. Animal Experiments

Animal study was performed in accordance with institutional guidelines of Research Ethics Committee of The Third Affiliated Hospital of Chongqing Medical University (General Hospital) (CQ-023-201601142). During the experiment, the pain of the animals was reduced as much as possible without affecting the results of the experiment. 4-week male BALB/c athymic nude mice bought from Shanghai Slac Laboratory Animal Company were used for in vivo tumor xenograft model. 5 × 10^6^ cells stably transfected with NC shRNA, TET1 shRNA, and *β*-catenin shRNA were injected subcutaneously in flanks of mice with 6 mice each group. After 30 days, mice were euthanized, and the weight of tumors was measured.

## 3. Results

### 3.1. TET1 Was Downregulated in Malignant Glioma Specimens

**Figure 1:**
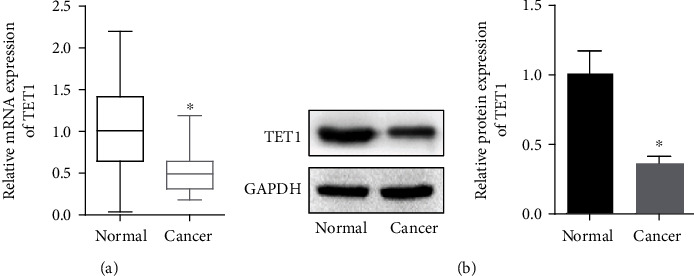
TET1 was downregulated in glioma specimens. (a) RT-qPCR was performed to detect the RNA level of TET1 in tumor tissues and the adjacent normal tissue of patients with glioma. (b) Western blot was adopted to analyses the expression of TET1. ^∗^*P* < 0.05.

To investigate the expression of TET1 in malignant glioma patients, we detected the relative RNA level of TET1 in WHO grade IV glioma specimens and adjacent normal tissues by RT-qPCR. The results showed the lower expression of TET1 in tumor tissues compared with adjacent normal tissues (Figure 1(a)). Additionally, results of Western blot confirmed the downregulation of TET1 in glioma specimens (Figure 1(b)).

### 3.2. Downregulation of TET1 Enhanced the Proliferation Ability of U251 and SW1783 Cells

**Figure 2:**
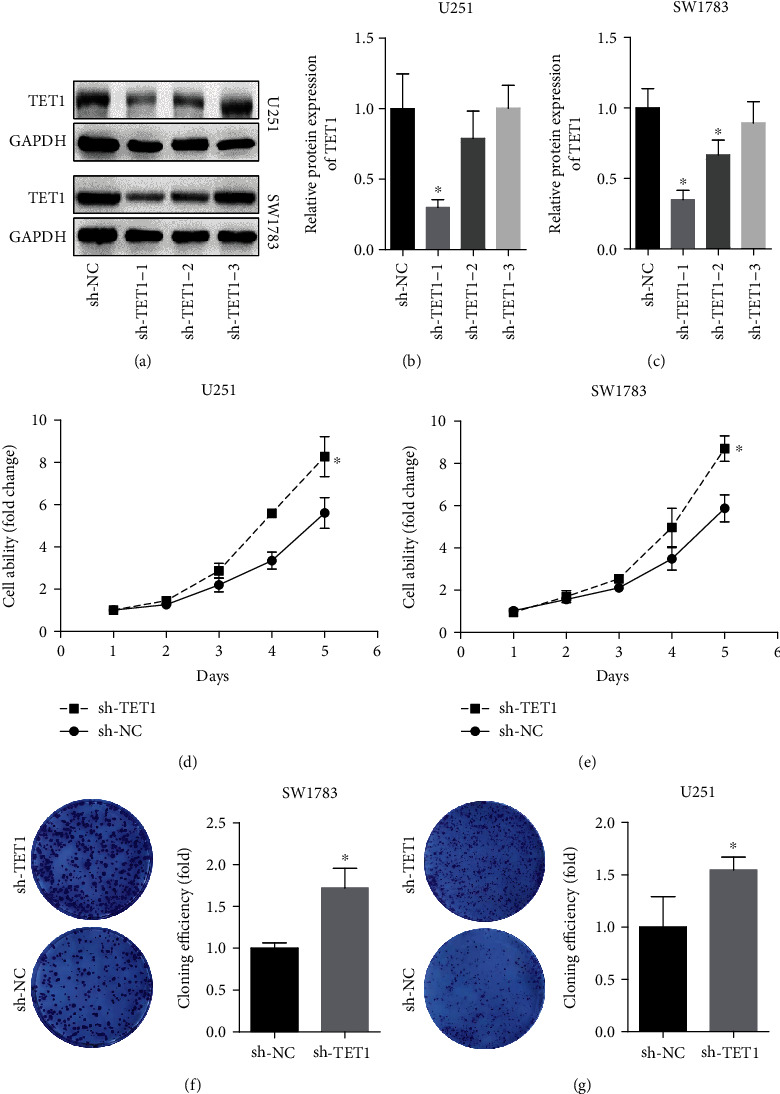
Downregulation of TET1 enhanced the proliferation ability of U251 and SW1783 cells. (a, b) The efficiency of TET1 shRNAs was detected by RT-qPCR. (b, c) CCK8 assay was performed to test the proliferation of U251 and SW1783 cells. (d, e) Colony formation assay was used to confirm the ability of cell proliferation. ^∗^*P* < 0.05.

To explore the roles TET1 played in tumor growth of glioma, we downregulated TET1 by transfecting glioma cells LN-229 and SW1783 with TET1 shRNA. Western blot was adopt to detect the efficiency of TET1 shRNAs (Figure 2(a)). Results of CCK8 assay showed the downregulation of TET1 could facilitate the proliferation of U251 and SW1783 cells (Figures 2(d) and 2(e)), and result of CFU showed that downregulation of TET1 could increase the colony formation of U251 and SW1783 cells (Figures 2(f) and 2(g)).

### 3.3. Downregulation of TET1 Promoted the Migration and Invasion of U251 and SW1783 Cells

**Figure 3:**
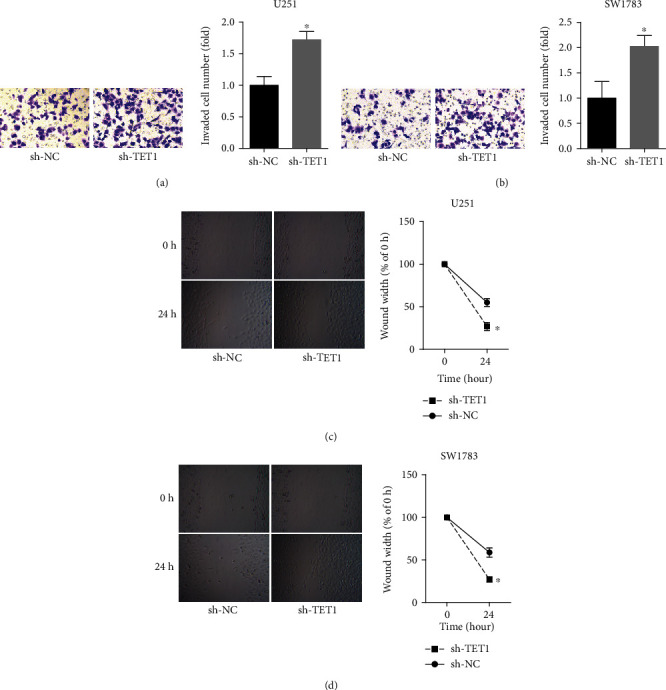
Downregulation of TET1 promoted the migration and invasion of U251 and SW1783 cells. (a, b) Transwell was used to detect the invasion of U251 and SW1783 cells. (c, d) Wound healing assay was adopted to determine the migration of U251 and SW1783 cells. ^∗^*P* < 0.05.

To test whether TET1 participates in the migration and invasion of U251 and SW1783 cells, wound healing assay and Transwell were performed. As shown Figures 3(a) and 3(b), downregulation of TET1 promoted the invasion of U251 and SW1783 cells. The inhibition of migration was tested after knock down of TET1 (Figures 3(c) and 3(d)).

### 3.4. Downregulation of TET1 Disrupted Cell Cycle Distribution and Inhibited Apoptosis of U251 and SW1783 Cells

**Figure 4:**
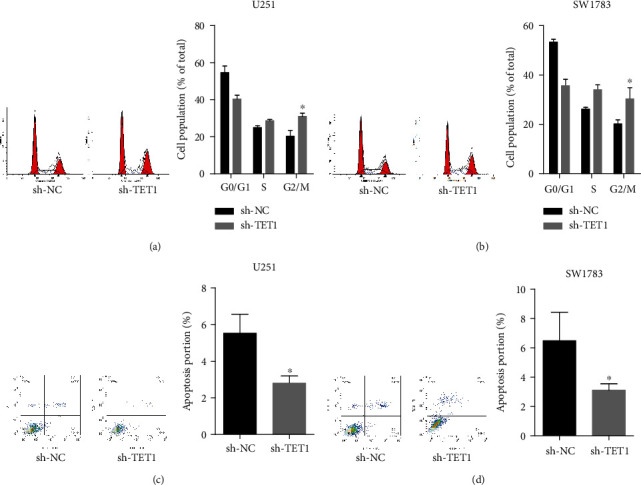
Downregulation of TET1 disrupted cell cycle distribution and inhibited apoptosis of U251 and SW1783 cells. (a, b) The cell cycle distributions of U251 and SW1783 cells were evaluated by flow cytometry. (c, d) The apoptosis rates of U251 and SW1783 cells were detected by flow cytometry. ^∗^*P* < 0.05.

To identify the function of TET1 in cell cycle distribution and cell apoptosis, we downregulated TET1 in U251 and SW1783 cells and performed flow cytometry. As shown in results, deficiency of TET1 induced the G1 cell cycle arrest and enhanced the ratio of G2 cells (Figures 4(a) and 4(b)). And the apoptosis of U251 and SW1783 cells was reduced (Figures 4(c) and 4(d)).

### 3.5. TET1 Deficiency Promoted Proliferation of U251 and SW1783 Cells In Vivo

**Figure 5:**
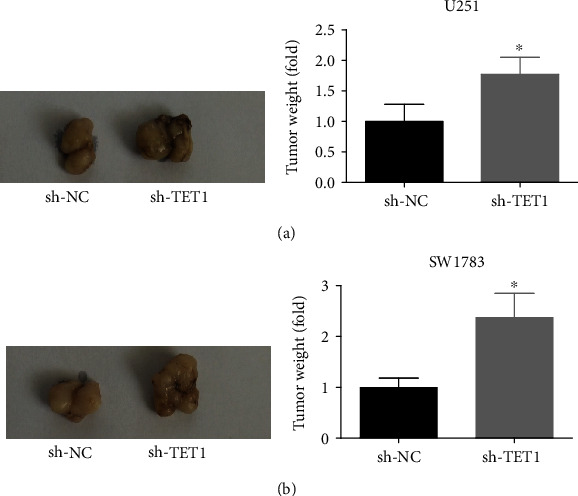
TET1 deficiency promoted proliferation of U251 and SW1783 cells in vivo. (a, b) Nude mice were injected with U251 and SW1783 cells which were transfected with negative control shRNA and TET1 shRNA. The tumors were excised after 30 d, and the weights were calculated. ^∗^*P* < 0.05.

Knockdown of TET1 could promote the cell proliferation, migration, and invasion in vitro. To detect its function on tumor growth in vivo, we stably transfected the U251 and SW1783 cells with negative control shRNA and TET1 shRNA and injected these cells subcutaneously into nude mice. As shown in Figures 5(a) and 5(b), knockdown of TET1 accelerated the growth of tumors.

### 3.6. TET1 Contributed to Glioma Cell Growth by Targeting Wnt/*β*-Catenin Pathway

**Figure 6:**
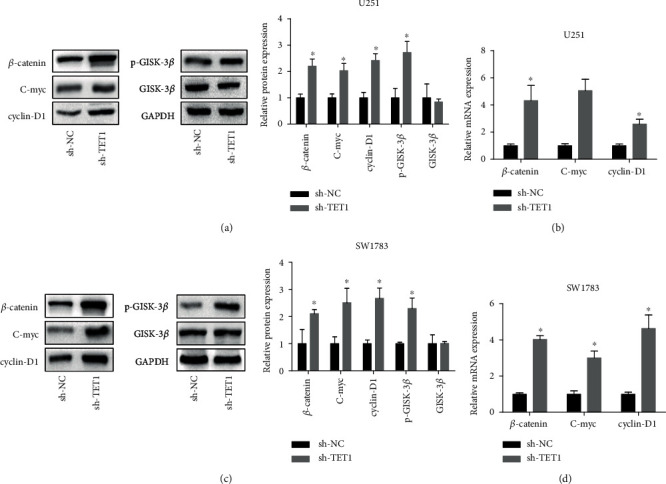
TET1 contributed to glioma cell growth by targeting Wnt/*β*-catenin pathway. (a) Western blot was adopted to detect the expression of key protein of Wnt pathway and its downstream targets. (b) RT-qPCR was taken to calculate the RNA level of *β*-catenin and its underlying targets in U251 cells. (a) Western blot was adopted to detect the expression of key protein of Wnt pathway and its downstream targets in SW1783 cells. (b) RT-qPCR was taken to calculate the RNA level of *β*-catenin and its underlying targets in SW1783 cells. ^∗^*P* < 0.05.

To investigate the molecular mechanisms targeted by TET1 in tumor growth of glioma, we adopted Western blot to detect the expression of cancer-related proteins. A previous study determined that TET1 acted as a tumor suppressor in nasopharyngeal carcinoma via antagonizing Wnt/*β*-catenin pathway [14].Corresponding with previous research, our work identified that downregulation of TET1 could increase the expression of key proteins of Wnt/*β*-catenin including *β*-catenin and phosphorylated GSK3*β* and the underlying targets of Wnt pathway such as cyclinD1 and c-myc (Figures 6(a)–6(c)). In addition, the enhancement in RNA level of *β*-catenin downstream target genes like cyclinD1 and c-myc was observed (Figures 6(b)–6(d)).

### 3.7. Downregulation of *β*-Catenin Inhibited Cell Migration and Invasion

**Figure 7:**
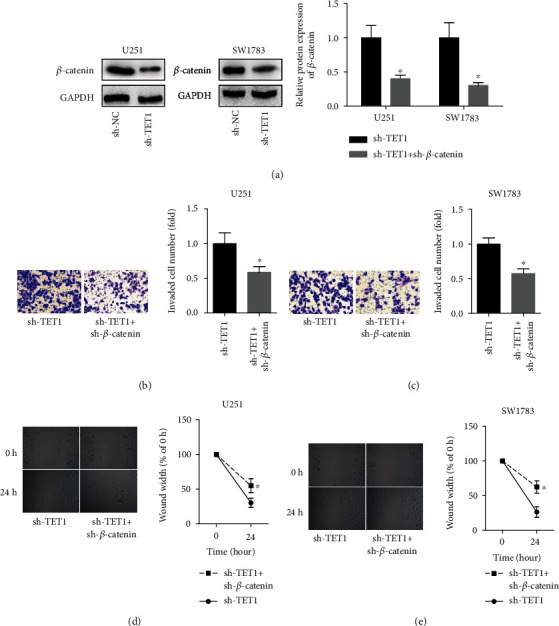
Downregulation of *β*-catenin inhibited cell migration and invasion. (a) Western blot and RT-qPCR were used to validate the efficiency of *β*-catenin shRNA. (b, c) Transwell experiments were adopted to determine the invasion of U251 and SW1783 cells. (d, e) Wound healing assay was taken to calculate the migration of U251 and SW1783 cells. ^∗^*P* < 0.05.

To confirm the role of Wnt/*β*-catenin pathway played in tumor suppression mediated by TET1, we downregulated the TET1 and *β*-catenin at the same time. The efficiency of *β*-catenin shRNA was determined by RT-qPCR (Figure 7(a)). Results of Transwell experiments showed that downregulation of *β*-catenin could inhibit the invasion of U251 and SW1783 cells (Figure 7(b)). Furthermore, results of wound healing assay showed that downregulation of TET1 reduced the migration of U251 and SW1783 cells (Figure 7(c)).

### 3.8. Downregulation of *β*-Catenin Inhibited Cell Growth In Vivo

**Figure 8:**
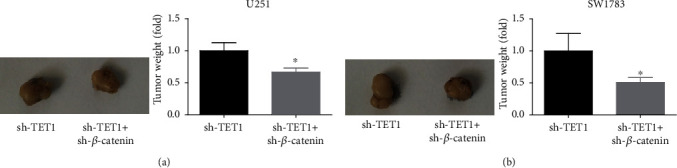
Downregulation of *β*-catenin inhibited cell growth in vivo. (a, b) U251 and SW1783 cells transfected with TET1 shRAN and cotransfected with TET1 shRNA and *β*-catenin shRNA were injected into nude mice. After 30 d, tumors were excised, and the weight of tumors was calculated. ^∗^*P* < 0.05.

Rescue experiments in vivo were performed to further confirm the critical the Wnt/*β*-catenin pathway played in tumor suppressor caused by TET1 deregulation. The weight of tumors were detected, and the results testified that downregulation of *β*-catenin could inhibit cell growth of U251 and SW1783 cells in vivo which was promoted by deficiency of TET1 (Figures 8(a) and 8(b)).

## 4. Discussion

It is well known that glioma is a malignant cancer with poor prognosis and low five-year survival rate [15]. FLAIRectomy can indicate the location of tumor invasion during surgery, increase tumor resection rate, and prolong patient survival. This may be a new direction for future surgery [16, 17]. However, although new methods and drugs are continuously applied in the clinic, the overall survival rate of patients with malignant glioma is still very low. To find new therapy target, great attention is paid on the investigation of molecular mechanisms for glioma progression [18, 19]. It is proved that alteration in p53, IDH gene, and neurofibromatosis 1 gene and genes in RTK pathways and Wnt/*β*-catenin pathway are appeared in glioma patients [20–23]. Research identifies that epigenetic alteration including aberrant DNA methylation, histone modification, and chromatin remodeling, and abnormal expression of microRNA and long-noncoding RNA played a critical role in the progression of glioma [24]. In our work, we identify the importance of TET1 in the tumorgenesis of glioma. It is found that the deficiency of TET1 in glioma tissues and downregulation of TET1 in U251 and SW1783 cells promoted cell proliferation, migration, and invasion and affected the distribution of cell cycles.

TET1 is one of the ten-eleven translocation (TET) family which could convert 5mC to 5hmC. Numerous studies have demonstrated the participation of TET1 in the pathogenesis of cancers, and most of the researches are in keeping with our results. Orr et al. testify the association between TET proteins and malignant glioma. It is found that 5hmC and TET genes are significantly reduced in human glioblastoma, and the low expression of TET gene is associated with poor prognosis [25]. In addition, Fu et al. find that TET1 is downregulated in glioma specimens, and the expression of TET1 is negatively related with malignant level of glioma. Moreover, they find the autophagy level in glioma is regulated by TET1 and deficiency of TET1 in U251 cells inhibits the autophagy and then promotes the proliferation and invasion of U251 cells [13]. However, there are several studies in contrast to our study. Takai et al. declare that they find 5hmC is markedly enriched in primary glioblastoma cells, and this enrichment is mediated by TET1 [26]. This may be on account of the difference in cell culture conditions and tumor types.

It well known that Wnt/*β*-catenin pathway is involved in a lot of biological process like cell proliferation, cell cycle distribution, and metabolism [27]. A mount of studies demonstrate that ectopic activation of Wnt pathway is observed in many kinds of cancers [28, 29], and downregulation of *β*-catenin inhibits the glioma growth [30, 31]. For instance, FBXO16 is discovered as a tumor suppressor in glioblastoma by targeting Wnt signaling pathway. FBXO16 overexpressing in RANG-2 cells shows increased degradation of *β*-catenin and markedly reduction of cell proliferation and migration caused by cycling 1 and c-myc which were downstream targets of *β*-catenin [32]. Upregulation of YAP promotes glioma cell proliferation via *β*-catenin activation [33]. In YAP knockdown experiments, level of *β*-catenin is decreased, and cell proliferation is inhibited [33]. Additionally, in nasopharyngeal carcinoma (NPC) cells, overexpression of TET1 suppresses cell growth of NPC and significantly inhibits the expression of *β*-catenin [14]. In this study, concomitant decrease in the level *β*-catenin and its downstream target protein cyclinD1 and c-myc are observed in TET1 knockdown experiments. In the rescue experiments, we downregulate TET1 and *β*-catenin at the same time. It is found that downregulation of *β*-catenin inhibits migration and invasion of glioma cells which are promoted by downregulation of TET1. The further experiments in vivo confirmed that TET1 inhibits glioma cell growth by targeting Wnt/*β*-catenin pathway.

In conclusion, our work demonstrates that deficiency of TET1 can promote the tumorgenesis of glioma by upregulating the expression of *β*-catenin. In addition, overexpression of *β*-catenin leads to markedly reduction of glioma cell growth which is enhanced by overexpression of TET1. Our work provides theoretical foundation for serving TET1/Wnt axis as a new target for treatments of malignant glioma patients.

## Data Availability

The datasets analyzed during the current study are available from the corresponding author on reasonable request.
